# The Chemopreventive Role of β-Elemene in Cholangiocarcinoma by Restoring *PCDH9* Expression

**DOI:** 10.3389/fonc.2022.874457

**Published:** 2022-07-12

**Authors:** Qing Wu, Xintong Shi, Yating Pan, Xinyi Liao, Jiahua Xu, Xiaoqiang Gu, Wenlong Yu, Ying Chen, Guanzhen Yu

**Affiliations:** ^1^ Department of Oncology, Longhua Hospital Affiliated to Shanghai University of Traditional Chinese Medicine, Shanghai, China; ^2^ Department of Biliary Tract Surgery, Shanghai Eastern Hepatobiliary Surgery Hospital, Naval Medical University, Shanghai, China; ^3^ Department of Gastroenterology, Changhai Hospital, Naval Medical University, Shanghai, China

**Keywords:** elemene, cholangiocarcinoma, cancer interruption, PCDH9, cell viability

## Abstract

**Background:**

β-Elemene, an effective anticancer component isolated from the Chinese herbal medicine Rhizoma Zedoariae, has been proved to have therapeutic potential against multiple cancers by extensive clinical trials and experimental research. However, its preventive role in cholangiocarcinoma (CCA) and the mechanisms of action of β-elemene on CCA need to be further investigated.

**Methods:**

A thioacetamide (TAA)-induced pre-CCA animal model was well-established, and a low dosage of β-elemene was intragastrically (i.g.) administered for 6 months. Livers were harvested and examined histologically by a deep-learning convolutional neural network (CNN). cDNA array was used to analyze the genetic changes of CCA cells following β-elemene treatment. Immunohistochemical methods were applied to detect β-elemene-targeted protein PCDH9 in CCA specimens, and its predictive role was analyzed. β-Elemene treatment at the cellular or animal level was performed to test the effect of this traditional Chinese medicine on CCA cells.

**Results:**

In the rat model of pre-CCA, the ratio of cholangiolar proliferation lesions was 0.98% ± 0.72% in the control group, significantly higher than that of the β-elemene (0. 47% ± 0.30%) groups (*p* = 0.0471). Kyoto Encyclopedia of Genes and Genomes (KEGG) pathway analysis showed that the top 10 pathways affected by β-elemene treatment were associated with energy metabolism, and one was associated with the cell cycle. β-Elemene inactivated a number of oncogenes and restored the expression of multiple tumor suppressors. PCDH9 is a target of β-elemene and displays an important role in predicting tumor recurrence in CCA patients.

**Conclusions:**

These findings proved that long-term use of β-elemene has the potential to interrupt the progression of CCA and improve the life quality of rats. Moreover, β-elemene exerted its anticancer potential partially by restoring the expression of PCDH9.

## Introduction

Cholangiocarcinoma (CCA) is a lethal epithelium malignancy characterized by late diagnosis and poor outcomes. The incidence and mortality of CCA, especially intrahepatic CCA, are on the rise worldwide. The only curative strategy for CCA is the complete removal of the primary tumor. However, up to 80% of CCA patients are diagnosed with an advanced, irresectable disease and unresectable CCA. Prognosis in these unresectable CCA is extremely poor, ranging from 6 to 12 months (1). Due to limited therapeutic options, early detection and chemopreventative strategies would be able to yield some favorable results, such as extended survival time (2). Ongoing investigations have proved that a number of potential drugs have been debated as effective chemopreventive agents to reduce cancer incidence. Recently, existing drugs such as aspirin, metformin, tamoxifen, and other alternatives have been shown to reduce cancer risk in a variety studies (3). Among these alternative medicines, traditional Chinese medicine (TCM) plays an important role in cancer prevention and interception (4–6).

TCM has long been used to maintain health and treat ailments. Growing evidence supports the utilization of TCM, alone or combined with other treatments, in reducing side effects associated with radio- and chemotherapy, improving the quality of life, lowering the risk of recurrence, and prolonging overall survival (OS) (7). Among these TCMs, β-elemene (β-1-methyl-1-vinyl-2,4-diisopropenyl-cyclohexane) is an active constituent of turmeric derived from Chinese herbal medicine Rhizoma Zedoariae and has been listed in the medical practices by State Administration of Pharmacy of China and Ministry of Health People’s Republic of China. β-Elemene has now been shown to have therapeutic potential in disease progression revealed *in vitro*, *in vivo*, and in clinical studies (8–10). For example, β-elemene could suppress tumor growth and angiogenesis, induce apoptosis and autophagy, and inhibit invasion and metastasis. Mechanically, β-elemene could suppress proliferative signaling, such as MAPK and PI3K/Akt/mTOR pathway, induce cell death, upregulate growth suppressors, deactivate invasion and metastasis-related genes, and attenuate angiogenesis by targeting VEGF (11, 12). All these findings support β-elemene as an effective agent for cancer treatment. However, whether β-elemene could be used as a chemopreventive drug needs to be explored more. Given the multi-targeting biological and molecular regulation of β-elemene, we try to explore β-elemene as a candidate chemopreventive agent in lowering cancer risk and interrupting cancer progression.

A reproducible animal model for pre-CCA (cholangiofibrosis) is important for exploring therapeutic and chemoprevention strategies for human CCA. An oral thioacetamide (TAA)-induced model of rat cholangiofibrosis (intrahepatic cholangial lesion preceding the development of CCA) was well-established (13). In this model, multifocal bile ductular proliferation was observed by the 9th week of TAA administration, and cholangiofibrosis (originally defined as intestinal-type CCA) was evident at the 16th week and diffusely distributed at the 22nd week (14). A conferred treatment before this time point might be helpful to inhibit the malignant transformation of cholangiolar ductal epithelium. Therefore, in this study, after 3 months of administrating rats with TAA, we treated these rats with a low dose of β-elemene for 6 months. We aimed to investigate the safety and efficiency of long-period utilization of β-elemene in treating TAA-induced pre-CCA. As expected, β-elemene reduced liver lesions induced by TAA, and interestingly, the life quality of experimental rats was greatly improved. The further *in vitro* cell line experiments offered evidence for an inhibitive effect of β-elemene on CCA cells and their derived tumors. We also identified genes that were significantly upregulated by RNA profiling, especially *PCDH9*.

*PCDH9* gene encodes the protein named protocadherin-9 (PCDH9), which is known as a tumor suppressor in glioma (15), hepatocellular carcinoma (16), and ovarian cancer (17). However, the role of PCDH9 in CCA was not identified. Extensive studies have reported that *PCDH9* gene expression was silenced by microRNAs, such as miR-215-5p and miR-589-3p in glioma cells (15, 18) and miR-200a-3p in ovarian cancer cells (17). In this study, β-elemene drove *PCDH9* gene expression, which further promoted cell apoptosis. Hence, our study explored the molecular mechanism of β-elemene exerting a suppressive effect on cancer cells, which is much closer to the clinical significance of basic research.

## Methods and Materials

### Animal Models

**Figure 1:**
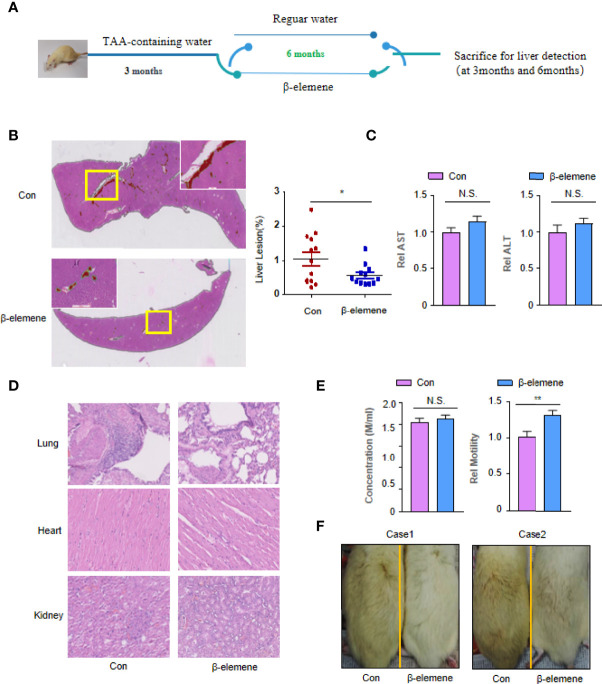
The effectiveness and safety of rats following long-term utilization of β-elemene. **(A)** Flowchart of the animal experiments. **(B)** Representative H&E staining image of liver lesions (red) of rats treated with or without β-elemene recognized by deep learning convolutional neural networks (CNNs). The ratio of cholangiolar proliferation lesions recognized by artificial intelligence (AI) was 0.98% ± 0.72% in the control group, significantly higher than that of the β-elemene (0. 47% ± 0.30%) group (*p* = 0.0471). Scale bar, 4 mm. **(C)** The serum alanine aminotransferase (ALT) and aspartate aminotransferase (AST) levels were measured in indicated mice. **(D)** No microscopic pathological changes in internal organs including heart, lung, and kidney in the β-elemene group compared with the control group. Scale bar, 800 μm. **(E)** The sperm motility, not concentration, of rats administered with β-elemene was significantly higher than that of mice not administered with β-elemene (*p* = 0.0033). **(F)** Aging phenotype assay. The control group was more yellow and shed more hair than the group treated with β-elemene. *p < 0.05; **p < 0.01; NS, not significant.

All experimental procedures were approved by the Experimental Animal Ethics Committee of Longhua Hospital Affiliated with the Shanghai University of TCM, China. Rats were housed as previously described (14). This experiment enrolled 20 adult male Sprague–Dawley (SD) rats (300–370 g), which were administered 300 mg/L of TAA (Cas: 27366-72-9, Hangyu Pharmaceutical Technology Development Co. Ltd., Changzhou, China) in their drinking water daily for 3 months. These rats were then divided into two groups: a control group (n = 10) and an experimental group (n = 10). The experimental group was intragastrically (i.g.) administered 20 mg/kg of β-elemene in 2 ml of solvent (Dalian Jingang Pharmaceutical Co. Ltd., Dalian, China) every day, whereas the control group was administered 2 ml of regular water. Three animals in each group at 3 months and all the others at 6 months were sacrificed, and their livers were harvested to examine the oncogenic effect of TAA and the preventive effects of β-elemene. This experiment detected whether β-elemene could interrupt the progression of CCA, as described in **Figure 1**.

### Histological Evaluation

The rats were anesthetized with pentobarbital sodium (40 mg/kg, intraperitoneally), and their livers were excised and assessed for diseases. The liver samples were then washed with saline solution and cut into 3~5-mm sections, half of which was stored at −80°C, and the other half was fixed in 4% paraformaldehyde for further histopathological examination. The lesions in each tissue section were carefully observed and evaluated by two individuals (GY and YC).

We developed an automated lesion detection framework based on convolutional neural networks (CNNs) to detect the liver lesions objectively and quantitatively as previously described (13).

### Cell Proliferation

QBC-939 and ICC-9810 cell lines were purchased from the Cell Center of the Chinese Academy of Sciences and certified without mycoplasma contamination by PCR. All these cells were maintained in Dulbecco’s Modified Eagle Medium (DMEM) with 10% fetal bovine serum (FBS) (Invitrogen, Carlsbad, CA, USA) and were cultured at 37°C in 5% CO_2_. These cells were seeded in 96-plates and treated with different concentrations of β-elemene for 24, 48, and 72 h. At indicated time points, two cells in each culture condition were collected, cell proliferation assay was measured using the CCK8 assay (Dojindo, Kumamoto, Japan), and IC50 was calculated (19). In detail, the absorbance of the solution was read spectrophotometrically at 450 nm with a reference at 650 nm using a microtiter plate reader (Becton-Dickinson, Franklin Lakes, NJ, USA), which was used as an indicator of cell viability.

### Apoptosis Assay

Flow cytometry analyses for cell death were performed with an annexin V-fluorescein isothiocyanate (FITC)/propidium iodide (PI) kit as described before (20). After treatment, cells were trypsinized, collected by centrifugation, washed with phosphate-buffered saline (PBS), and resuspended at a density of 5 × 10^5^ cell/ml with 1× annexin V binding buffer. Then 5 μl of annexin V-FITC conjugates and 5 μl of PI solution were added and incubated for 15 min in the dark. Finally, cells were incubated with 1× annexin V binding buffer and analyzed within 1 h by flow cytometric analysis (BD FACS Aria SORP, BD Biosciences, San Jose, CA, USA). At least 20,000 cells were analyzed to determine the percentage of apoptotic cells.

### Cell Cycle Analysis

The cells were starved by serum-free medium for 24 h for synchronization at the G0 phase. Next, the cells were incubated with fresh medium supplemented with 10% serum for an additional amount of time (from zero to 24) and collected every 2 or 4 h. After being washed with PBS, the cells were fixed in ice-cold 70% EtOH for 2 h at 4°C, centrifuged at 2,000 rpm for 5 min, and washed with PBS twice. The cells were then incubated in RNase solution (1 mg/ml) at 37°C for 30 min and stained with PI (50 μg/ml) for cell cycle analysis (488-nm excitation). A Gallios Flow Cytometer (Beckman, Brea, CA, USA) was used for pulse processing and collecting cells fluorescing above an emission wavelength of 620 nm, and data were processed with Modfit software (Verity Software House, Topsham, ME, USA).

### cDNA Microarray

Total mRNA from QBC-939 cells treated with or without β-elemene (800 μg/ml) for 24 and 48 h were collected using TRIZOL Reagent (Invitrogen Life Technologies). RNA purity and integrity were determined by NanoDrop ND-1000 and denaturing agarose gel electrophoresis. The qualified RNA was then used for cDNA microarray analysis (Affymetrix^®^ Human Genome U219 Array Plate) as previously reported (21). The ratios for the signal intensities of Cy3:Cy5 of each spot >1.5 and <0.67 were defined as the cutoff benchmarks to identify upregulated and downregulated genes. Gene ontology analysis was performed to determine the most significantly altered genes or pathways (22).

### Tissue Specimens and Immunohistochemical Study

Four-micron-thick paraffin-embedded sections of CCA samples (n = 49) were prepared (23) and processed for immunohistochemistry analysis. Antibody against PCDH9 was purchased from Sigma-Aldrich, Inc. (St. Louis, MO, USA) (dilution, 1:100; R05779). A streptavidin-biotin kit (#KIT-9720; Maixin-Bio, Fuzhou, China) was used to visualize antibody binding to the tissue sections. Two individuals (GY and YC) independently evaluated all samples using an Olympus CX31 microscope (Olympus Optical). The final results were compiled using a semiquantitative scoring system as previously described (24, 25).

### Statistics

All statistical analyses were performed using SPSS software (Chicago, IL, USA) and Prism software (version 7.0; GraphPad Software). Categorical data were analyzed using χ^2^ statistics tests. The Kaplan–Meier method was used to estimate survival rates, and the Cox proportional hazards model for multivariate survival analysis was used to assess predictors related to survival. A *p*-value <0.05 was considered statistically significant.

## Results

### β-Elemene Interrupted Tumor Formation Induced by Thioacetamide

The main characteristic of the liver lesions was cholangiofibrosis, a kind of cholangial lesion preceding the development of CCA. To test whether β-elemene exerted its suppressive effect on liver lesions and CCA initiation, we designed an experimental schedule (**Figure 1A**). Three months after β-elemene treatment, none of the 3 rats treated with β-elemene had cholangiolar proliferation, while 2 of 3 rats untreated with β-elemene presented multifocal bile ductular proliferation (**Figure S1**). Six months later, all rats untreated with β-elemene presented cholangiolar proliferation or cholangiofibrosis, while only 2 of 6 showed visible cholangiolar proliferation or cholangiofibrosis (**Figure S2**). An automated lesion detection framework based on deep learning CNNs clearly showed the location and quantities of liver lesions (red) in whole slide images (WSIs) (**Figure 1B**). The results showed a representative image of liver lesions of rats treated with or without β-elemene displayed by artificial intelligence (AI). The ratio of cholangiolar proliferation lesions recognized by AI was 0.98% ± 0.72% in the control group, which was significantly higher than that of the β-elemene (0. 47% ± 0.30%) group (**Figure 1B**) (*p* = 0.0471). These data showed for the first time that β-elemene could interrupt the development of pre-CCA induced by TAA. Of note, as a kind of TCM, β-elemene did not have disadvantages on the normal function of liver metabolism (**Figure 1C**).

### Safety and Quality of Life for Long-Term Utilization of β-Elemene

One rat was suffocated when i.g. administered with β-elemene. There were no instances of TAA- or drug-related mortality during the 9-month period. Six-month use of β-elemene did not result in any visible microscopic pathological changes in internal organs including the heart, lung, and kidney compared with the control group (**Figure 1D**). However, two more findings were observed in this experiment. The first one is that the sperm motility, not concentration, of rats administered with β-elemene was significantly higher than of those not administered with β-elemene (**Figure 1E**). The other one is that, probably due to aging, rats in the control group moved slower, became listless, were more yellow, and shed more hair than those with β-elemene treatment (**Figure 1F**). These data provide some evidence that long-term utilization of β-elemene is relatively safe and might improve the life quality of the rats exposed to carcinogens.

### β-Elemene Induces Apoptosis and Cell Cycle Arrest of Human Cholangiocarcinoma Cells and Aberrant Gene Expression

**Figure 2:**
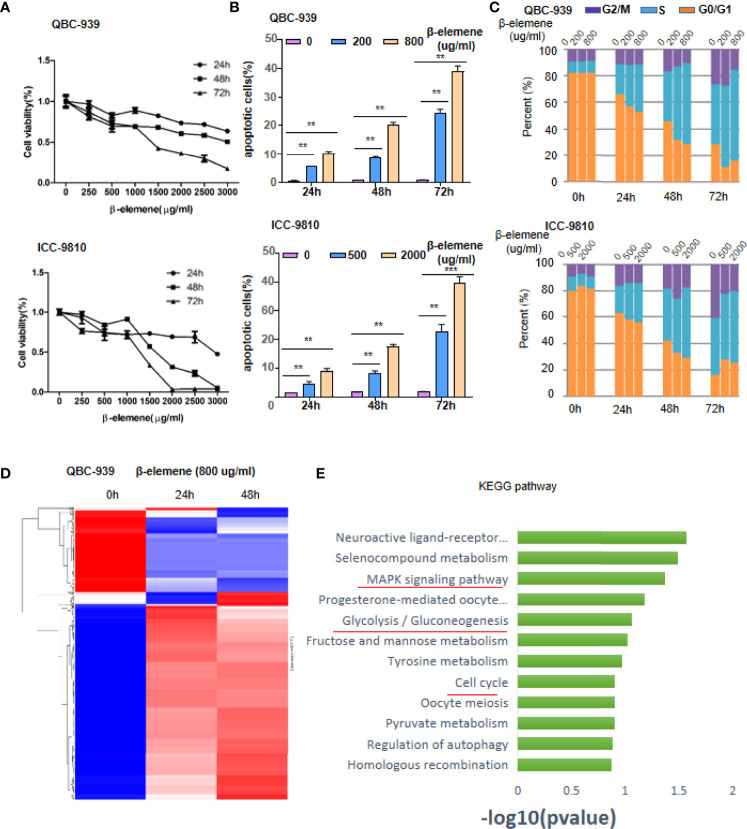
β-Elemene induces apoptosis, cell cycle arrest of human cholangiocarcinoma (CCA) cells, and aberrant gene expression. **(A)** Cell viability of QBC-939 and ICC-9810 was measured by Trypan blue staining assay following β-elemene treatment at indicated concentrations and time points. **(B)** Apoptotic QBC-939 or ICC-9810 cells were tested by propidium iodide (PI)/annexin V double staining kit following β-elemene treatment at indicated concentrations and time points. **p < 0.01; ***p < 0.001. **(C)** Cell cycle analysis. QBC-939 or ICC-9810 cells were administered with indicated concentrations and time points of β-elemene treatment. Then, cells were collected for PI staining and fluorescence-activated cell sorting (FACS) analysis. **(D, E)** QBC-939 cells were treated with β-elemene (500 μg/ml) for 24 or 48 h and subjected to cDNA array analysis, and differentially expressed genes (fold change >1.5 or <0.67, *p* < 0.05) are shown in panel **(D)** Significant Kyoto Encyclopedia of Genes and Genomes (KEGG) pathway retrieved by clusters of genes involved in the intersection of two time points **(E)**.

In accordance with previous studies (12, 26), our data confirmed that β-elemene treatment inhibits cell proliferation of both CCA cell lines, QBC-939 and ICC-9810, in a dose- and time-dependent manner *in vitro*, although there are a little differences between these two cells (**Figure 2A**). Next, we selected two concentrations of β-elemene to treat QBC-939 and ICC-9810 cells. As shown in **Figure 2B**, with the increase in β-elemene concentration and time, the apoptosis of CCA cells also increased gradually (**Figures 2B**, **S3**). Consistently, cell cycle assay proved that β-elemene would induce CCA cells arresting in the S phase, indicating that β-elemene plays a suppressive role of tumor cells probably by blocking DNA replication (**Figure 2C**). To identify the regulatory circuit of β-elemene in CCA cells, QBC-939 cells were treated with 500 μg/ml and subjected to a cDNA array assay. After β-elemene treatment (500 μg/ml) for 24 h, a total of 1,746 genes were differentially expressed (1,207 upregulated and 539 downregulated). At the time point of 48 h, a total of 2,216 genes were differentially expressed (1,459 upregulated and 757 downregulated) (**Figure 2D**). When analyzed by gene ontology, the top 10 Kyoto Encyclopedia of Genes and Genomes (KEGG) pathways were as follows: neuroactive ligand-receptor interaction, selenocompound metabolism, MAPK signaling pathway, progesterone-mediated oocyte maturation, glycolysis/gluconeogenesis, fructose and mannose metabolism, tyrosine metabolism, cell cycle, oocyte meiosis, and pyruvate metabolism (**Figure 2E**).

### β-Elemene Induces Aberrant Expression of Invasion/Metastasis-Related Genes, Especially *PCDH9*

**Figure 3:**
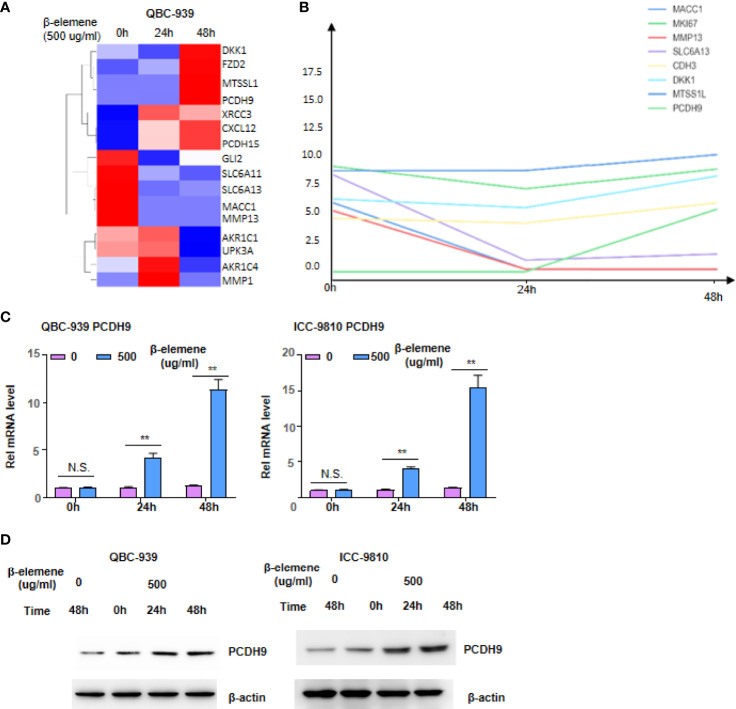
β-Elemene induces aberrant expression of invasion/metastasis-related genes, especially *PCDH9*. **(A, B)** Cluster of changed genes involved in tumor invasion and metastasis **(A)** and the linear trend of selected genes at indicated time points **(B)**. **(C, D)** qRT-PCR **(C)** and Western blotting **(D)** analyzed PCDH9 in QBC-939 and ICC-9810 cells following β-elemene treatment at indicated concentrations and time points. NS, not significant; **p < 0.01.

Given that KEGG pathways have not presented the significant genes regulated by β-elemene, several significant genes associated with energy metabolism and invasion/migration were selected for cluster analysis (**Figures S4A**, **3A**). Among all the genes associated with cell metabolism, those associated with Aldo-Keto Reductase gradually decreased when β-elemene was continuously administered, while the genes associated with gluconeogenesis, including PCK2 and TPI, gradually increased (**Figure S4B**). Among the genes associated with invasion/migration, those associated with metastasis promotion, such as MMP1, MMP13, and SLC6A11, were decreased, while the genes associated with metastasis suppression, including PCDH9 and MTSSL1, were increased when β-elemene was continuously administered (**Figure 3B**). Two of these genes, PCDH9 and PCK2, were validated using RT-PCR, which was consistent with the cDNA array results (**Figures 3C**, **S4C**). Of note, Western blotting confirmed the consistent trend of PCDH9 expression induced by β-elemene depending on both time and dose (**Figure 3D**). Inactivation of tumor suppressors and activation of oncogenes contribute malignant transformation of human tumors. Our data provide a clue that β-elemene is able to activate tumor suppressors and inactivate oncogenes simultaneously.

### PCDH9 Depletion Impaired the Effect of β-Elemene on Cholangiocarcinoma Cells

**Figure 4:**
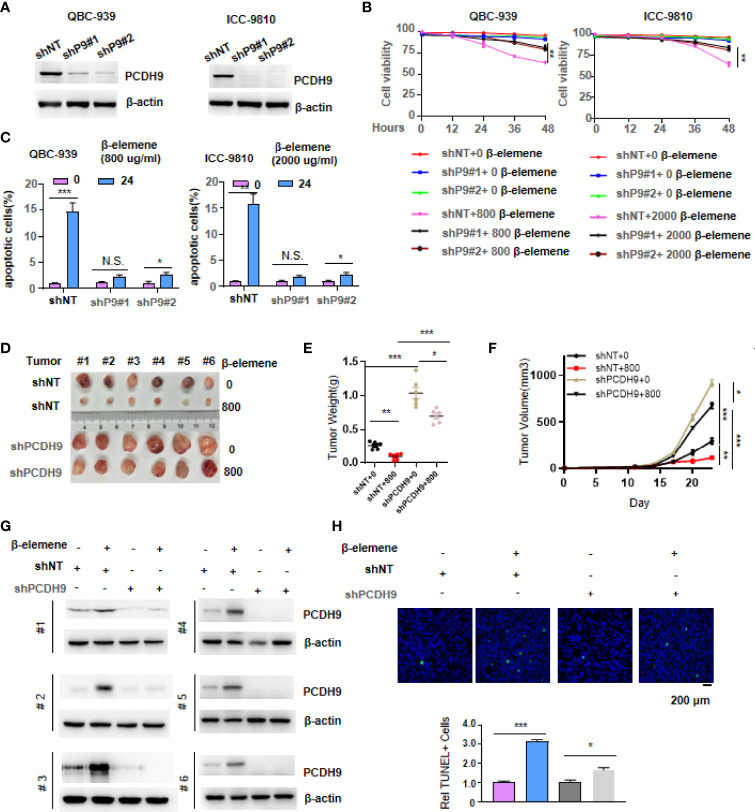
PCDH9 depletion impaired the effect of β-elemene on cholangiocarcinoma (CCA) cells. **(A)** PCDH9 was stably depleted by two independent shRNAs, and Western blotting tested the efficiency of knocking down PCDH9. **(B)** Cell viability of both genetically manipulated QBC-939 and ICC-9810 cells was measured by Trypan blue stain assay following β-elemene treatment at indicated concentrations and time points. **p < 0.01. **(C)** Apoptotic assay of both genetically manipulated QBC-939 and ICC-9810 cells was performed by propidium iodide (PI)/annexin V double staining kit following β-elemene treatment at indicated concentrations and time points. * *p* < 0.05; **p < 0.01; ***p < 0.001; NS, not significant. **(D–F)** The stable cell lines constructed in panel A were transplanted into the left groin of nude mice (n = 6, each group). Solid tumors were dissected and imaged at the end of the experiment **(D)**. Tumor weight was measured, *p < 0.05, **p < 0.01, ***p < 0.001 **(E)**, and tumor volume was recorded every 3 days, *p < 0.05, **p < 0.01, ***p < 0.001 **(F)**. **(G)** Western blotting analysis of PCDH9 expression in solid tumors derived from cells in panel **(A)**. **(H)** TUNEL staining was performed to examine apoptotic cells in solid tumors. Scale bar, 100 μm, *p < 0.05; ***p < 0.001.

To ascertain the role of PCDH9 in CCA, especially under the condition of with or without β-elemene treatment, we knocked down *PCDH9* gene in both QBC-939 and ICC-9810 cells with two independent small hairpin RNAs (**Figure 4A**). Then, we detected the cell viability of these genetically manipulated cells under β-elemene treatment. As shown in **Figure 4B**, the cell viability dramatically increased after depleting PCDH9 in QBC-939 or ICC-9810 cells once β-elemene treated these cells, indicating that PCDH9 is a major gene upregulated by β-elemene (**Figure 4B**). Apoptosis assay showed a decrease of apoptotic cells after PCDH9 depletion under β-elemene (**Figures 4C**, **S5**). Thus, the CCA cells without PCDH9 expression are much more sensitive to β-elemene treatment.

To certify the function of β-elemene at the animal level, we transplanted these genetically manipulated QBC-939 cells into the groin of nude mice. Once the tumors reached 0.2 cm^2^, we treated these mice every day with or without β-elemene for a week. After 23 days, we excised tumors from nude mice and imaged solid tumors. Loss of PCDH9 reinforced tumor formation, and the treatment of β-elemene has no significant inhibition on tumor formation, while the transplantation of QBC-939 control cells formed a smaller tumor once upon treatment with β-elemene (**Figure 4D**). We also measured the tumor weight and tumor growth trend, both of which offered consistent results. We observed that when we subjected these mice to β-elemene treatment, the tumor derived from QBC-939 control cells almost stopped growing, while the tumor without PCDH9 continued to grow (**Figures 4E, F**). We randomly tested PCDH9 expression in three groups of xenograft tumors and obtained consistent expression of PCDH9 within cell lines, proving that the solid tumors were derived from genetically manipulated QBC-939 cells in **Figure 4A** (**Figure 4G**). Furthermore, immunohistochemistry (IHC) was performed using an antibody against TUNEL, which is indicative of apoptotic cells, in solid tumors (**Figure 4H**).

Hence, the above data were consistent with a previous report that described the suppressive role of PCDH9 in tumor cells and PCDH9 depletion sensitized CCA cells to β-elemene.

### Expression of PCDH9 in Patients With Cholangiocarcinoma Showed Clinical Significance

**Figure 5:**
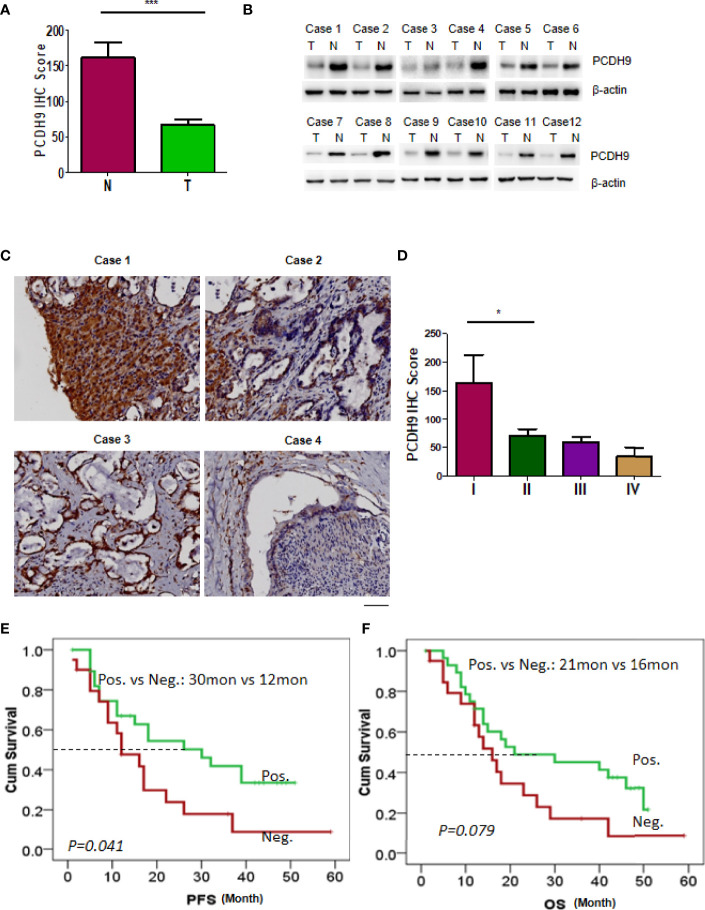
Expression of PCDH9 in patients with cholangiocarcinoma (CCA) showed clinical significance. **(A)** Immunohistochemistry (IHC) was used to detect the expression of PCDH9 protein in the normal liver tissue and CCA tissue. The IHC intensity was scored, and the statistical data is shown ***p < 0.001. **(B)** Twelve paired CCA and normal tissue were analyzed by Western blotting using antibodies against PCDH9. **(C)** Representative patterns of PCDH9 protein in CCA samples were displayed by IHC. Scale bar, 400 μm. **(D)** CCA samples were analyzed by IHC and scored for four grades. The statistical data are shown *p < 0.05. **(E)** Patients with PCDH9 expression had a longer time to recur than those without PCDH9 expression (median PFS: 32 vs 12 months, *p* = 0.041). **(F)** Patients with PCDH9 expression had a longer overall survival (OS) as compared with those without PCDH9 expression (median OS: 21 vs 16 months, *p* = 0.079).

We first detected PCDH9 protein level in rats’ liver lesions treated with or without β-elemene. The level of PCDH9 mRNA was increased in rats’ liver lesions following β-elemene treatment (**Figure S6A**). Next, we detected PCDH9 expression profiles in human CCA. Intensive staining of PCDH9 was observed in normal liver, which could be used as an internal control (**Figure S6B**). Strong staining of PCDH9 was also observed in the cytoplasm and nuclear of cholangiocytes of normal bile ducts (**Figure S6C**). To statistically clarify the intensity of PCDH9 in normal and tumor tissue, we collected 49 CCA samples. The score of PCDH9 IHC staining in normal cells was 55.4 ± 25.0, significantly higher than that in tumor cells, 32.3 ± 30.4 (*p* = 0.0155) (**Figure 5A**). We selected 12 paired CCA tumors and normal liver tissue for Western blotting analysis. As shown in **Figure 5B**, in each paired sample, the PCDH9 expression level was heavily downregulated (**Figure 5B**). We showed four representative images of PCDH9 IHC staining (**Figure 5C**) and further calculated the intensity of four stages of CCA tumor; meanwhile, the statistical results showed that the intensity of PCDH9 decreased with the increase of stage of CCA (**Figure 5D**).

The median cumulative OS in this small cohort of CCA patients was 16 months (range, 1–59 months). The Kaplan–Meier analyses revealed that patients with PCDH9-negative tumors had a higher probability of recurrence as compared with those with PCDH9-positive tumors (median progression-free survival (PFS): 12 vs 30 months, *p* = 0.041; **Figure 5E**). Although not reaching a significant value, patients with negative PCDH9 expression had a short OS as compared with those with positive PCDH9 expression (median OS: 16 vs 21 months, *p* = 0.079; **Figure 5F**). Subsequent multivariate analysis using the Cox regression model did not demonstrate PCDH9 as an independent indicator of PFS in patients with CCA. Taken together, PCDH9 expression in CCA patients showed a good sign of prognosis.

## Discussion

**Figure 6:**
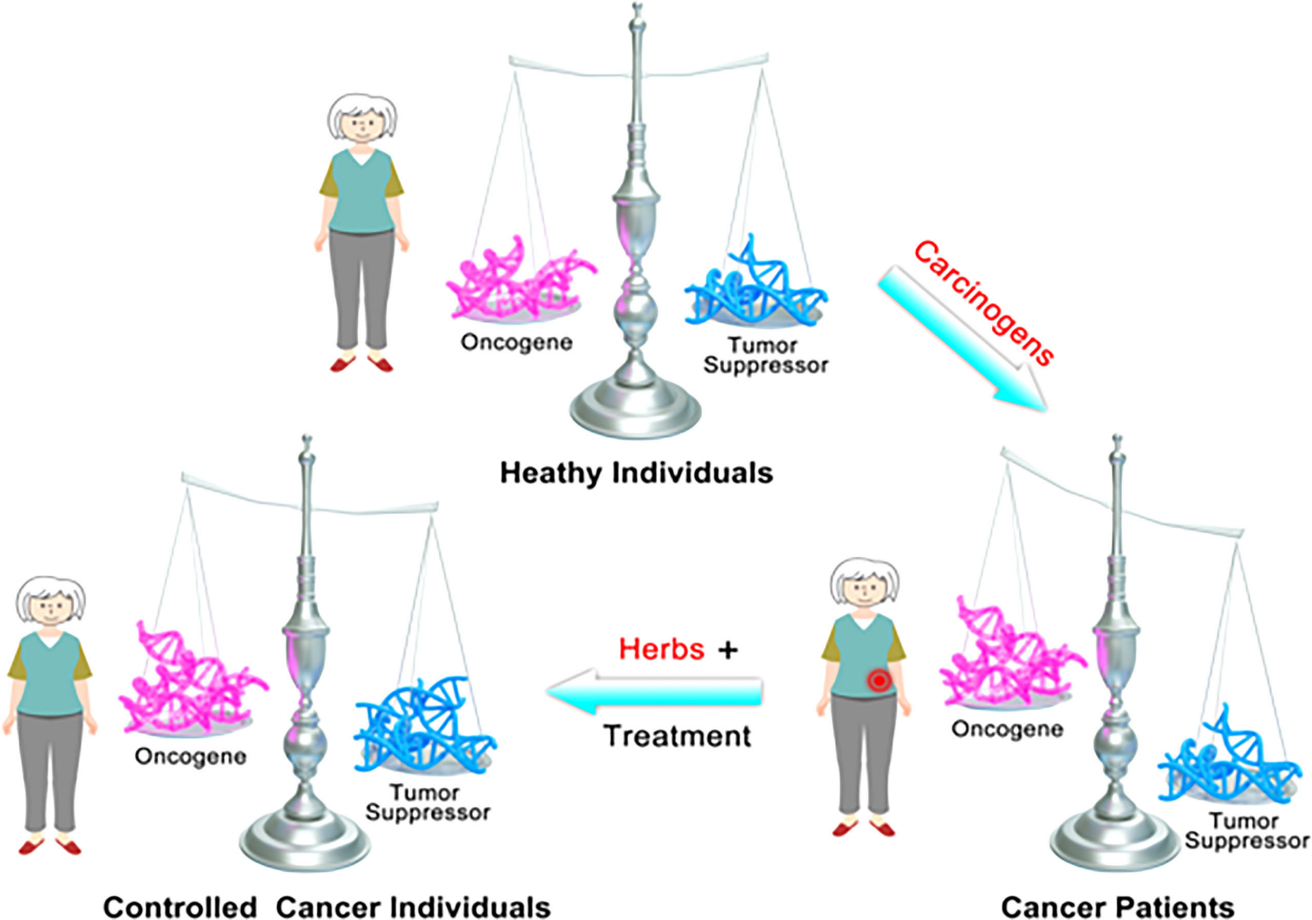
Schematic diagram shows the mechanism underlying long-term use of β-elemene in cancer treatment or cancer interruption.

The most valuable finding in this study is the fact that long-term utilization of β-elemene is able to interrupt the progression of pre-CCA and probably improves the life quality of rats. In detail, β-elemene exerted its anticancer potential *via* upregulating invasion/metastasis suppressor and inactivating genes associated with invasion/metastasis. These novel findings demonstrated some of the unique properties of β-elemene as an herbal agent for cancer treatment and cancer interruption; especially, β-elemene could strengthen healthy qi to eliminate pathogenic factors, an aim of TCM treatment (**Figure 6**).

Cancer incidence and mortality rates for some cancers have been increasing at an alarming speed in China (27). Given that most kinds of cancers have certain common causes, delaying, reversing, or inhibiting cancer insurgence or cancer progression is important (3). In addition to avoiding carcinogens and maintaining a healthy lifestyle, drug repurposing could offer an effective preventive approach to reduce cancer risk, including aspirin and metformin (3). In this study, we established a realistic scenario mimicking individuals with extensive carcinogen exposure: rats were administered with TAA for 3 months, and then this procedure terminated. Utilizing this model, we provided first-hand evidence that β-elemene is a good candidate as a chemopreventive agent. First, long-term use of β-elemene did not bring significant side effects, and β-elemene increases the motility of sperm in rats and inhibits the characteristics of aging of rats, both of which are our focus. The second is the fact that the liver lesions by TAA induction became less following β-elemene administration, indicating that β-elemene did actively interrupt the cancer development process before a tumor mass dispersed in the liver. Although the mechanism of β-elemene improving life quality remains unclear, all the data indicate that β-elemene has some advantages of preventing cancer and potentially improving life quality.

The scientific basis for developing β-elemene as an effective adjuvant agent for cancer treatment is its potential in targeting the hallmarks of cancer: suppressing proliferative signaling, inducing cell death, upregulating growth suppressors, deactivating invasion and metastasis, etc. (11) Our cDNA array results partially reproduced those molecular mechanisms of the anticancer activities of β-elemene. However, unlike chemo- or radiotherapy, which mainly targets cell cycle and DNA synthesis (28), KEGG pathway analysis showed that half of the top 10 pathways affected by β-elemene treatment were associated with cancer metabolism, especially gluconeogenesis, and only one was associated with the cell cycle. Multiple metabolism-associated genes were induced or inhibited by β-elemene treatment. For example, two Aldo-Keto Reductases, AKR1C1 and AKR1C4, were downregulated, while two involved in gluconeogenesis, TPI and PCK2, were upregulated by β-elemene treatment. These data provide a novel clue for investigating the mechanism of β-elemene as an agent for cancer treatment. However, the exact mechanism underlying β-elemene modulating cancer metabolism needs further investigation. As a well-studied Chinese medicine, another impressive result is that β-elemene treatment not only led to inactivating invasion and metastasis, characterized by decreased expression of MMP1 and MMP13, but also led to accelerating growth suppressors and invasion/metastasis suppressors. A previous study revealed that β-elemene could reduce epithelial–mesenchymal transition (EMT)-associated nuclear transcription factors Snail1/2, Twist, and SIP1 (29). These EMT-associated biomarkers were not listed in our cDNA array result of β-elemene treatment. However, multiple metastasis suppressors, such as Dickkopf-1 (DKK1) (30), FZD2 (31), and PCDH9 (25), were gradually increased along with continuous β-elemene treatment. This is supposed to be a unique property of TCM, which leads to the turnover of malignant transformation of cancer cells. However, the detailed epigenetic mechanism on how β-elemene suppressed the EMT-driving factors and activated multiple metastasis suppressors is still elusive and deserves to be clarified.

PCDH9 is a metastasis regulator, which is well-described in gastric cancer metastases in our previous study (25). Here, we detected its expression pattern in CCA and tried to explore its prognostic value. Non-cancerous liver cells and bile duct epithelium showed positive staining of PCDH9, significantly higher than that in tumor cells. Moreover, patients with PCDH9-negative tumors have a higher probability of recurrence than those without PCDH9 expression. Therefore, our results confirmed PCDH9 as a tumor suppressor and a predictor of patients’ survival time. Restoring PCDH9 expression by β-elemene treatment is a major reason for the use of β-elemene in cancer therapy and interruption. However, the mechanisms underlying β-elemene-induced activity of PCDH9 need further investigation. For example, we should prepare the rats with specifically PCDH9 knockout in the bile duct, and then TAA-induced pre-CCA animal will be applied to test whether PCDH9 depletion in the bile duct would block CCA occurrence and progression.

In conclusion, there are several novel findings of anticancer actions of β-elemene treatment, including interrupting cancer progression, improving life quality, regulating cancer metabolism, and accelerating metastasis suppressors. However, whether β-elemene can be used as a chemopreventive agent needs a large high-risk population cohort-based clinical trial. The novel mechanisms underlying β-elemene treatment also should be further investigated. Overall, efforts to unravel the complex signal pathways will facilitate β-elemene to become a truly successful therapeutic or preventive strategy for CCA.

## Data Availability Statement

The datasets presented in this study can be found in online repositories. The names of the repository/repositories and accession number(s) can be found in the article/**Supplementary Material**.

## Ethics Statement

The studies involving human specimens were reviewed and approved by the Institutional Ethics Committee of Shanghai Eastern Hepatobiliary Surgery Hospital, Naval Medical University. The patients/participants provided their written informed consent to participate in this study. The animal study was reviewed and approved by the Institutional Ethics Committee of Longhua Hospital Affiliated with the Shanghai University of Traditional Chinese Medicine.

## Author Contributions

Conception/design: GY, QW, and YC. Acquisition of data: QW, XS, YP, XL. Animal models: XS, YP, and XL. Data analysis and interpretation: JX, QW, and XG. Tissue specimens: XS and WY. Source: GY, XS, YC, and QW. Manuscript writing: GY and YC. Final approval of manuscript: GY and YC. All authors listed have made a substantial, direct, and intellectual contribution to the work and approved it for publication.

## Funding

This research was supported by the projects from the National Natural Science Foundation of China (Nos. 81572856, 81972721, and 8217112952) and the National Thirteenth Five-Year Science and Technology Major Special Project for New Drug Innovation and Development: The construction of a demonstration technology platform for the clinical evaluation of new drugs for malignant tumor and other diseases (2017ZX09304001).

## Conflict of Interest

The authors declare that the research was conducted in the absence of any commercial or financial relationships that could be construed as a potential conflict of interest.

## Publisher’s Note

All claims expressed in this article are solely those of the authors and do not necessarily represent those of their affiliated organizations, or those of the publisher, the editors and the reviewers. Any product that may be evaluated in this article, or claim that may be made by its manufacturer, is not guaranteed or endorsed by the publisher.

## Supplementary Material

Supplementary Figure 1 β-elemene interrupted the formation of rat cholangiofibrosis induced by TAA at three months. At 3 months after ß-elemene administration, rats were sacrificed and liver lesions were examined. 2 of 3 rats in the group without β-elemene treatment (Mock) presented liver lesions (upper), while none of those treated with β-elemene treatment had visible liver lesions (lower). Scale bar, 200 μm.

Supplementary Figure 2 β-elemene interrupted the formation of rat cholangiofibrosis (intrahepatic cholangial lesion preceding the development of cholangiocarcinoma) induced by TAA at 6 months. At 6 months after ß-elemene administration, all rats were sacrificed and liver lesions were examined. 7 of 7 rats in the group without β-elemene treatment (Mock) presented liver lesions (upper), while 2 of 6 treated with β-elemene had visible liver lesions(lower). Scale bar, 200 μm.

Supplementary Figure 3 Apoptotic QBC-939 or ICC-9810 cells were tested by PI/Annexin V double staining kit following β-elemene treatment at indicated concentrations and time points. Representative image of apoptosis assay was shown and Q2 area indicated the percent of apoptotic cells in the group.

Supplementary Figure 4 Apoptotic genetically manipulated QBC-939 or ICC-9810 cells was performed by PI/Annexin V double staining kit following β-elemene treatment at indicated concentrations and time points. * P<0.05, ***P<0.001.

Supplementary Figure 5 β-elemene inhibited cell proliferation by regulating energy metabolism-related genes. **(A, B)** Cluster of changed genes involved in metabolism **(A)** and the linear trend of selected genes at indicated time points **(B)**. **(C)** QBC-939 cells were treated with β-elemene (500 ug/ml) for 48 hours and PCK2 mRNA levels were measured using quantitative RT-PCR.

Supplementary Figure 6 After TAA induction, rats were treated with or without β-elemene (500 ug/ml) for 1 week and then liver was obtained for quantitative RT-PCR using primers against rat PCDH9 **(A)**. **P<0.01. PCDH9 expression patterns in human liver **(B)** and bile ducts **(C)** were shown by IHC. Scale bar, 200 μm.

## Abbreviations

CCA, cholangiocarcinoma; QOL, quality of life; AI, artificial intelligence; PCDH9, protocadherin-9; WSIs, whole slide images.
