# Supplementary figures and images for: The Chemopreventive Role of β-Elemene in Cholangiocarcinoma by Restoring PCDH9 Expression
Source: Front Oncol. 2022 Jul 12;12:874457. doi: 10.3389/fonc.2022.874457 (PMC9314746; doi:10.3389/fonc.2022.874457)

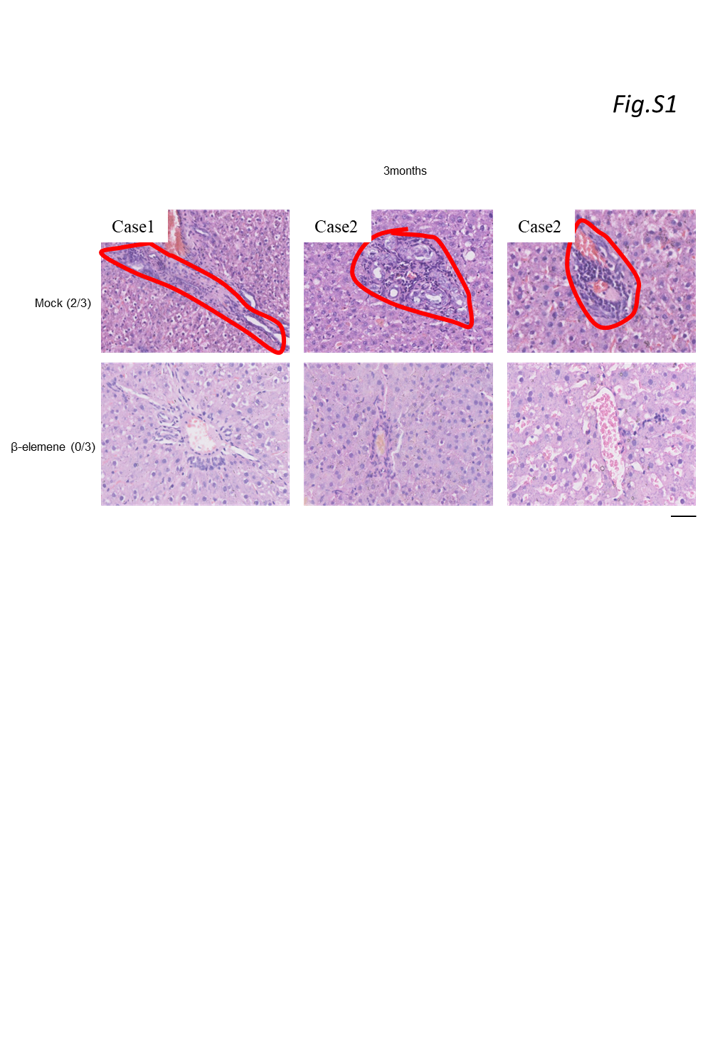

Supplement: Supplementary Figure 1 — β-elemene interrupted the formation of rat cholangiofibrosis induced by TAA at three months. At 3 months after ß-elemene administration, rats were sacrificed and liver lesions were examined. 2 of 3 rats in the group without β-elemene treatment (Mock) presented liver lesions (upper), while none of those treated with β-elemene treatment had visible liver lesions (lower). Scale bar, 200 μm. [file Image_1.tif]

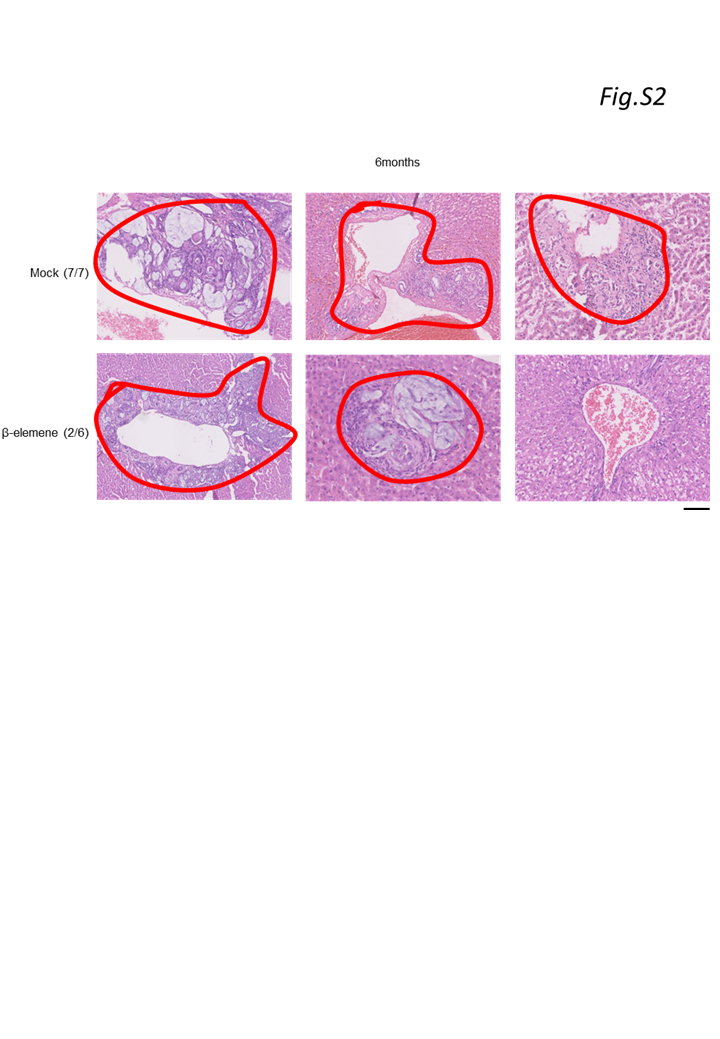

Supplement: Supplementary Figure 2 — β-elemene interrupted the formation of rat cholangiofibrosis (intrahepatic cholangial lesion preceding the development of cholangiocarcinoma) induced by TAA at 6 months. At 6 months after ß-elemene administration, all rats were sacrificed and liver lesions were examined. 7 of 7 rats in the group without β-elemene treatment (Mock) presented liver lesions (upper), while 2 of 6 treated with β-elemene had visible liver lesions(lower). Scale bar, 200 μm. [file Image_2.tif]

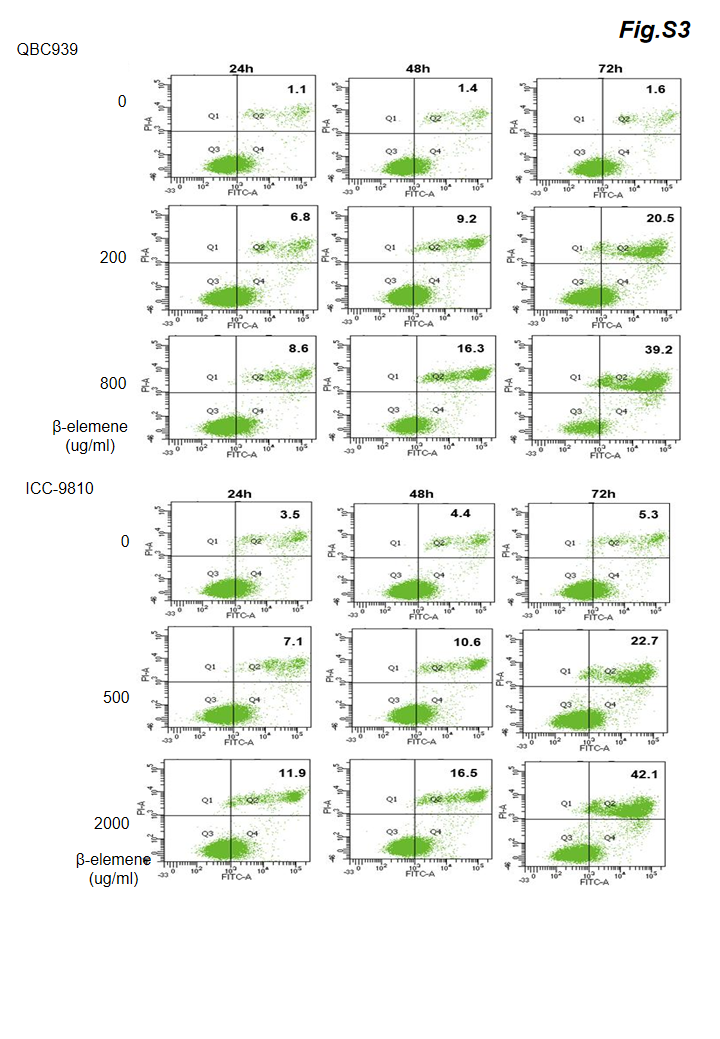

Supplement: Supplementary Figure 3 — Apoptotic QBC-939 or ICC-9810 cells were tested by PI/Annexin V double staining kit following β-elemene treatment at indicated concentrations and time points. Representative image of apoptosis assay was shown and Q2 area indicated the percent of apoptotic cells in the group. [file Image_3.tif]

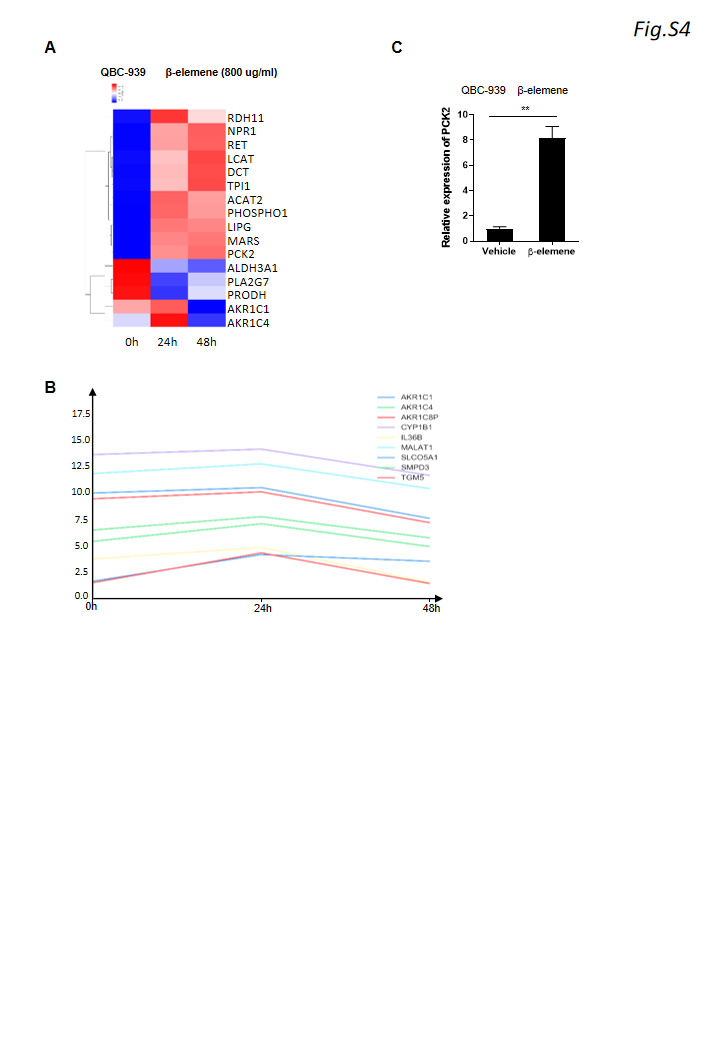

Supplement: Supplementary Figure 4 — Apoptotic genetically manipulated QBC-939 or ICC-9810 cells was performed by PI/Annexin V double staining kit following β-elemene treatment at indicated concentrations and time points. * P<0.05, ***P<0.001. [file Image_4.tif]

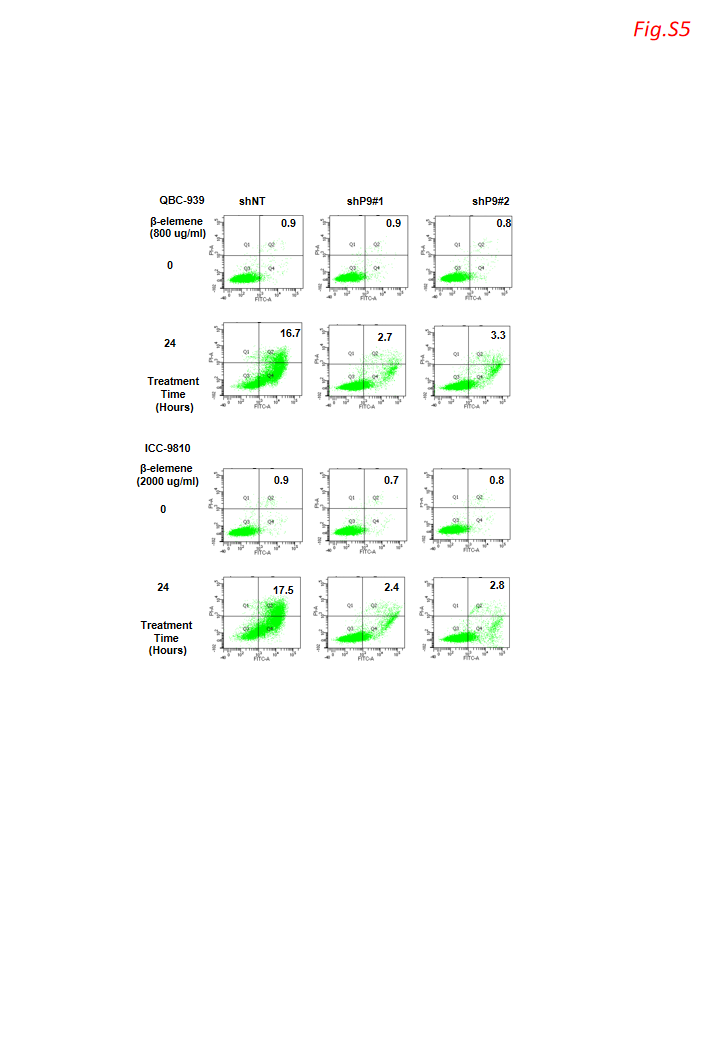

Supplement: Supplementary Figure 5 — β-elemene inhibited cell proliferation by regulating energy metabolism-related genes. (A, B) Cluster of changed genes involved in metabolism (A) and the linear trend of selected genes at indicated time points (B). (C) QBC-939 cells were treated with β-elemene (500 ug/ml) for 48 hours and PCK2 mRNA levels were measured using quantitative RT-PCR. [file Image_5.tif]

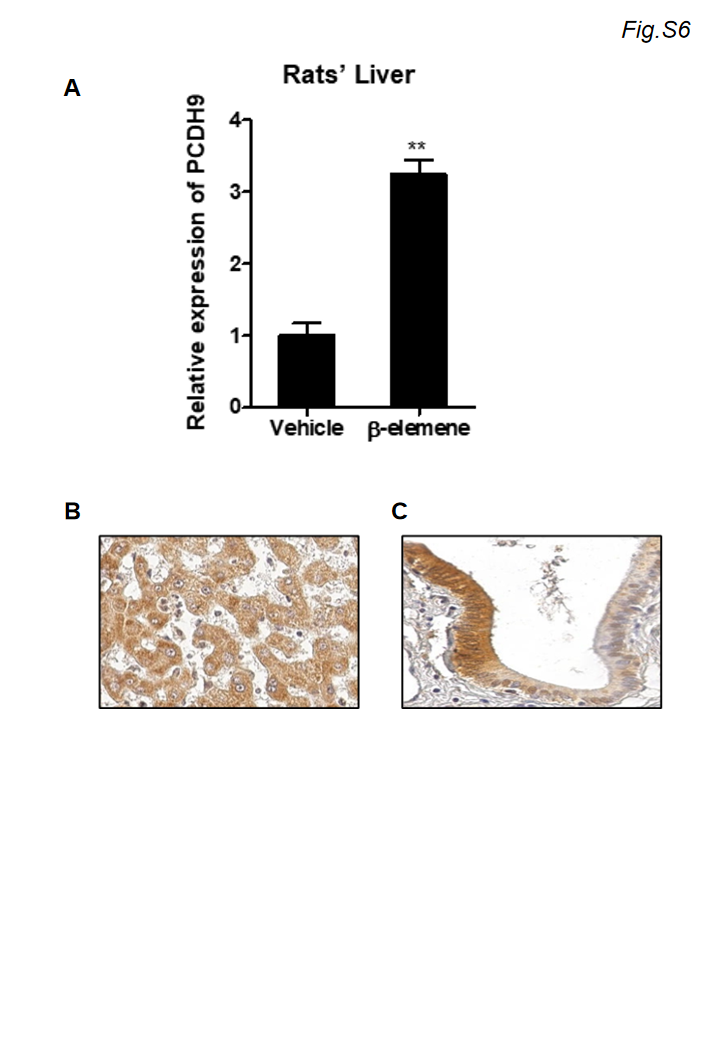

Supplement: Supplementary Figure 6 — After TAA induction, rats were treated with or without β-elemene (500 ug/ml) for 1 week and then liver was obtained for quantitative RT-PCR using primers against rat PCDH9 (A). **P<0.01. PCDH9 expression patterns in human liver (B) and bile ducts (C) were shown by IHC. Scale bar, 200 μm. [file Image_6.tif]
