# Supplementary material for: The Chemopreventive Role of β-Elemene in Cholangiocarcinoma by Restoring PCDH9 Expression
Source: Front Oncol. 2022 Jul 12;12:874457. doi: 10.3389/fonc.2022.874457 (PMC9314746; doi:10.3389/fonc.2022.874457)
Supplement: Supplementary file 7 [file DataSheet_1.pdf]

Age（1<60, 1	Gender（1, M	Size (1<3cm,	Invasion (0=	Nodal metast	Differentiat	TNM（1=I，2	PCDH9（0=n	Survival statu	Survival time	Recurrence（	Rucurrence status
1	2	1	3	1	3	2	0	0	9	7	0
2	2	1	3	1	2	2	0	0	23	17	0
1	2	1	3	1	2	2	0	1	12	12	1
1	1	2	3	1	2	4	0	0	14	12	0
1	2	1	3	1	3	2	1	0	6	5	0
2	1	2	3	1	2	3	1	0	10	5	0
1	1	1	3	1	2	2	1	3	1	1	1
1	1	2	3	1	2	3	1	0	40	32	0
1	2	1	2	1	2	2	1	1	48	48	1
2	2	2	3	1	2	3	1	0	8	6	0
1	1	1	0	0	1	1	1	1	43	43	1
1	2	2	3	0	2	3	0	0	26	22	0
1	2	2	3	0	2	3	0	0	5	5	0
1	2	2	2	1	3	2	1	0	50	47	1
1	2	2	3	1	2	4	1	0	11	5	0
2	1	1	3	1	3	2	0	0	16	11	0
1	2	1	1	0	2	1	1	1	47	47	1
2	2	2	3	0	2	3	0	0	12	9	0
1	2	2	3	0	2	3	0	3	2	2	1
2	2	2	3	0	1	3	1	1	51	51	1
2	1	2	3	1	2	3	1	0	9	7	0
1	2	2	2	1	2	2	0	3	36	36	1
1	1	2	3	0	1	3	1	1	42	42	1
1	1	2	3	1	3	3	0	0	6	5	0
1	1	1	3	0	2	2	1	1	49	49	1
2	1	2	3	1	2	3	0	0	29	26	0
2	1	2	3	0	2	3	1	0	46	39	0
1	2	2	3	1	3	3	0	0	2	2	0
2	1	2	3	1	3	3	1	0	21	18	0
1	2	1	1	0	2	1	0	1	59	59	1
2	1	2	3	0	2	3	1	0	19	18	0
1	2	2	3	1	1	3	1	1	44	44	1
1	1	1	3	1	1	2	0	0	5	1	0
1	2	2	3	1	2	3	1	0	15	11	0
1	2	2	2	1	2	2	0	0	13	12	0
1	1	1	3	0	1	2	0	0	17	16	0
1	1	1	3	1	1	2	1	0	30	26	0
2	2	2	3	1	3	3	1	0	18	15	0
1	1	2	3	1	3	3	1	1	12	12	1
1	1	2	3	1	2	3	1	0	5	5	1
1	1	2	3	1	3	4	1	0	14	11	0
1	2	2	3	1	1	3	1	1	51	30	0
1	1	1	3	0	2	2	0	0	12	9	0
1	1	2	3	1	2	3	1	0	12	7	0
1	1	1	3	1	3	2	1	0	9	6	0
2	1	1	3	0	3	2	1	0	42	39	0
1	1	1	3	1	2	4	0	0	18	17	0
2	2	2	3	0	2	3	0	0	42	37	0
1	2	2	3	1	2	3	1	0	14	14	1
